# Quad-Band Polarization-Insensitive Square Split-Ring Resonator (SSRR) with an Inner Jerusalem Cross Metamaterial Absorber for Ku- and K-Band Sensing Applications

**DOI:** 10.3390/s22124489

**Published:** 2022-06-14

**Authors:** Mohammad Lutful Hakim, Touhidul Alam, Mohammad Tariqul Islam, Mohd Hafiz Baharuddin, Ahmed Alzamil, Md. Shabiul Islam

**Affiliations:** 1 Pusat Sains Ankasa (ANGKASA), Institut Perubahan Iklim, Universiti Kebangsaan Malaysia (UKM), Bangi 43600, Selangor, Malaysia; p108762@siswa.ukm.edu.my; 2 Department of Computer Science and Engineering (CSE), International Islamic University Chittagong (IIUC), Kumira, Chattogram 4318, Bangladesh; 3 Department of Electrical, Electronic and Systems Engineering, Faculty of Engineering and Built Environment, Universiti Kebangsaan Malaysia (UKM), Bangi 43600, Selangor, Malaysia; hafizb@ukm.edu.my; 4 Electrical Engineering Department, College of Engineering, University of Hail, Hail 81481, Saudi Arabia; aa.alzamil@uoh.edu.sa; 5 Faculty of Engineering (FOE), Multimedia University, Persiaran Multimedia, Cyberjaya 63100, Selangor, Malaysia; shabiul.islam@mmu.edu.my

**Keywords:** metamaterial absorber, polarization-insensitive, quad-band, Ku- and K-band applications, sensing application

## Abstract

The development of metamaterial absorbers has become attractive for various fields of application, such as sensing, detectors, wireless communication, antenna design, emitters, spatial light modulators, etc. Multiband absorbers with polarization insensitivity have drawn significant attention in microwave absorption and sensing research. In this paper, we propose a quad-band polarization-insensitive metamaterial absorber (MMA) for Ku- and K-band applications. The proposed patch comprises two square split-ring resonators (SSRR), four microstrip lines, and an inner Jerusalem cross to generate four corresponding resonances at 12.62 GHz,14.12 GHz, 17.53 GHz, and 19.91 GHz with 97%, 99.51%, 99%, and 99.5% absorption, respectively. The complex values of permittivity, permeability, refractive index, and impedance of MMA were extracted and discussed. The absorption mechanism of the designed MMA was explored by impedance matching, equivalent circuit model, as well as magnetic field and electric field analysis. The overall patch has a rotational-symmetrical structure, which plays a crucial role in acquiring the polarization-insensitive property. The design also shows stable absorption for both transverse electric (TE) and transverse magnetic (TM) modes. Its near-unity absorption and excellent sensing performance make it a potential candidate for sensing applications.

## 1. Introduction

A metamaterial is a non-natural material structure that possesses rare material properties, such as negative permittivity, negative permeability, reverse doppler effect, and negative refractive index, known as metamaterial (MTM) properties [1]. MTM properties depend on the geometry of the unit cell structure with a stable structural composition. These extraordinary physical properties make MTMs appropriate for numerous applications, such as sensing [2,3], imaging [4], metamaterial coding [5], lensing [6], reflect arrays [7], terahertz applications [8], invisible clocks [9], antennae [10,11,12], absorbers [13], programable analog differentiators [14], etc. The perfect or near-perfect metamaterial absorber has the ability to absorb a specific frequency by preventing reflection and transmission of electromagnetic (EM) waves at a given frequency [15,16,17,18,19]. MTMs have attracted significant attention due to their extensive potential application areas, such as imaging, optical switching, energy harvesting, bolometry, radar cross-sectioning, antenna side-lobe reduction, SAR reduction, solar cells, sensing, etc. [20,21,22,23].

Therefore, MMAs can replace traditional absorbers, such as wedge-shaped, Salisbury screen, Jauman layer, and ferrite absorbers due to their bulkiness and thickness, although limited to a few applications [24]. The main benefit of MMAs over traditional absorbers is their ease of fabrication, low cost, lightweight, ultra-thin thickness, and near-unity absorption [25]. Most MMAs consists of a three-layer sandwich model (metal lossy substrate-metal) [26]. In MMAs, near-unity absorption peaks are achieved by controlling the imaginary and real part of complex dielectric, magnetic permeability and electrical permittivity. The input impedance of MMAs matches with free space impedance, which is achieved through specific geometrical design of the top metal (resonator) [27]. Impedance matching between air and the MMA reduces the reflected power at a particular frequency, and the bottom metal blocks EM wave transmission through the MMA [28]. The major limitations of the resonator base of MMAs are the narrow low values of the absorption band, which reduce the operating range and accuracy of the MMA. This limitation can be overcome by multi-band MMAs, which have an increased operating range and efficiency [14,29,30,31]. Multiband perfect absorption with full programmability of the absorbed bands was demonstrated in [14] by in situ tuning of an overmoded scattering system equipped with a programmable metasurface to the desired functionality. Moreover, polarization-insensitive behavior is also an important feature of MMAs, resulting in stable absorption properties at different polarization angles, which improves the usability of MMAs at different polarization values [29,32,33]. MMAs have the potential used for sensing applications in the microwave range. Various devices have been proposed for different sensing applications, such as permittivity sensors, refractive index sensors, grin sensors, density sensors, temperature sensor, and glucose sensors [29,30,32,33,34,35,36,37]. Moreover, K- and Ku-band frequencies can be applied for short-range microwave sensing [32,38,39].

Various metamaterial absorber is designed in the microwave (C, X, Ku, K) to the terahertz frequency band [40,41,42]. Ku and K band frequency has wide application in the radar, telecommunications, and sensor fields. A magnetic plasmon based metamaterial sensor has been designed in [43] for infrared wavelength, where the metamaterial was designed by Ag nanowire on Ag substrate. In [44], a splits ring resonator-based refractive index sensor is presented for protein sensing. A circular split-ring resonator (CSRR) metamaterial absorber was presented in [13] for K-band absorption and sensing applications, showing two 99.9% absorption peaks at 21.6 GHz and 24.04 GHz. The complete dimensions of the CSRR is 10 × 10 × 1.6 mm^3^. A quad-band wrenched-square-shaped resonator was proposed in [24], and a triple-band square split-ring resonator (SSRR) with an inner Jerusalem cross was presented in [45]. Both designs ([45] and [24]) achieved absorption peaks above 95% and exhibited polarization-insensitive behavior at S, X, and Ku frequency bands. In [46], a combination of eight identical 7-shapes and SSRR achieved three absorption peaks at 8.5, 13.5, and 17 GHz with 99.9%, 99.5%, and 99.9% absorption, respectively. In [47], A V-shaped polarization-insensitive MMA was designed for Ku- and K-band frequency applications, achieving absorption peaks at 15.52 and 27.24 GHz with 98.38% and 90.7% absorption, respectively. A T-shaped polyimide substrate-based polarization-insensitive MMA was presented in [48], and polarization-insensitive behavior was achieved because of the rotational symmetry of the MMA, with two absorption peaks at 16.77 GHz and 30.92 GHz with 98.7% and 99.3% absorption, respectively. In [49], a diagonally slotted patch MMA was designed for Ku-band applications, producing two absorption peaks at 12.45 and 14.18 GHz with 99.73% and 99.87% absorption, respectively. The orientation of the diagonal slot was 45°; due to this design, the MMA exhibited polarization-sensitive behavior. Most MMAs achieve single or dual absorption peaks at Ku- or K-band frequencies; some are polarization-sensitive and show lower absorption peaks or larger unit-cell sizes.

In this paper, we present an MMA for Ku- and K-band sensing applications. The geometry of the proposed MMA was chosen to provide quad-band polarization-insensitive absorption behavior. The designed MMA simulated for TM and TE modes, and all simulation setups resulted in similar absorption curves due to the symmetrical rotational design. We evaluated the proposed MMA in order to understand the effect of structural design on absorption behavior. In the following sections, we discuss the metamaterial properties, normalized input impedance, polarization conversion ratio (PCR), and H-field and E-fields with respect to absorption behavior. The advantages of the designed MMA include its quad-band absorption peaks with near-unity and polarization insensitivity.

## 2. Metamaterial Absorber Design

### 2.1. Unit Cell Design and Absorption Calculation

In his section, we discuss the design of an MMA unit cell with an absorption mechanism. The square split-ring resonator (SSRR) achieves quad-band near-unity absorption peaks. FR4 substrate materials with 1.6 mm thickness were selected for the absorber design due to their low cost, zero water absorption, and versatility, making them commercially attractive. The dielectric constant, thermal conductivity, and loss tangent of the substrate are 4.3, 0.3 W/K/m, and 0.025, respectively. Copper was used for the patch and ground design, with an electrical conductivity (ρ) of 5.96 × 10^7^ S/m.

**Figure 1:**
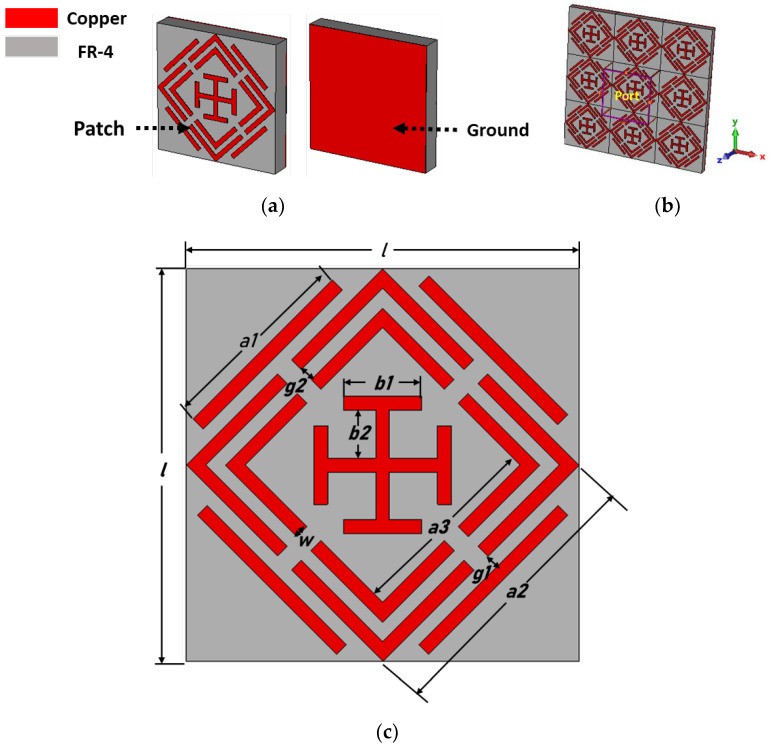
Patch design of the proposed MMA. (**a**) Perspective view, (**b**) simulation setup and (**c**) front view.

**Figure 2:**
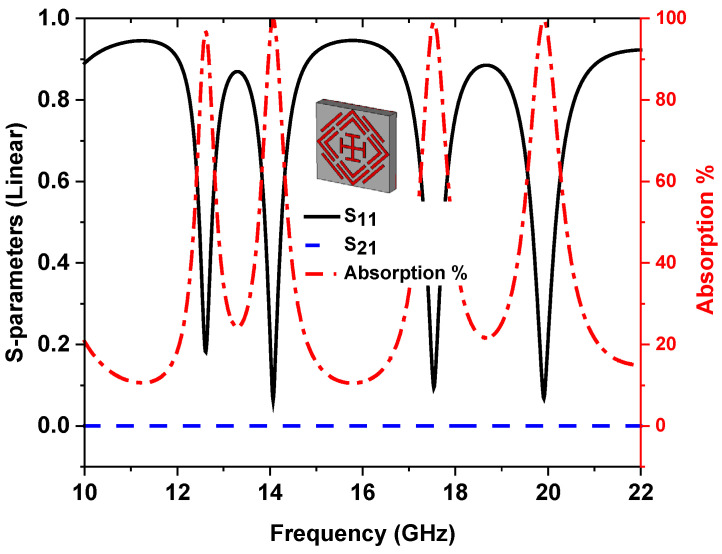
Absorption and s-parameter plot.

Figure 1 shows a front view of the MMA unit cell with a sketch of all required dimensions. The MMA patch design consists of a Jerusalem cross, two square split rings, and four microstrip lines. The unit cell dimensions are 10 × 10 × 1.6 mm^3^, and all the design parameters are recorded in Table 1. The proposed MMA was designed and simulated using the CST microwave studio [50], where the unit cell boundary conditions were applied along the Y- and X-axes, and electromagnetic waves were applied along the negative Z-axis. The absorption behavior A(ω) was determined according to Equation (1) [27].

(1) $$A(\omega )=1-S_{11}^{2}-S_{21}^{2}$$

where *S*_11_ and *S*_21_ are reflection and transmission coefficients, respectively, as shown in Figure 2, and four near-zero reflection coefficient (*S*_11_) resonance peaks are achieved at 12.62 GHz, 14.12 GHz, 17.53 GHz, and 19.91 GHz. A copper ground of 0.035 mm thickness results in a zero transmission coefficient (*S*_21_), which can be obtained by calculation of the skin depth [27].

(2) $$\mathrm{Skin}\;\mathrm{depth},\;\delta =\sqrt{\frac{\rho }{\pi f\mu }}$$

where permeability (μ) is 1, resistivity (ρ) is 1.72Ω−m, with lower frequency defined as f=12.62GHz. The skin depth becomes δ=0.0065 mm, which completely blocks the electromagnetic (EM) wave transmission through the MMA. Therefore, Equation (1) becomes:

(3) $$A(\omega )=1-S_{11}^{2}$$

The peak 97% absorption at 12.62 GHz and 99% absorption at 14.12 GHz, 17.53 GHz, and 19.91 GHz were attained for the proposed MMA presented in Figure 2. The high-quality (*Q*) factor represents high sensitivity, where the *Q* factor is calculated by *Q* = *fc/FWHM*, where *f_C_* is the center frequency, and *FWHM* is the full wave of half maximum [51]. The *Q* factors of the proposed MMA at 12.62 GHz, 14.12 GHz, 17.53 GHz, and 19.91 GHz are 39.43, 34.43, 37.29, and 34.32, respectively.

### 2.2. Evaluation of MMA and Metamaterial Property Analysis

**Figure 3:**
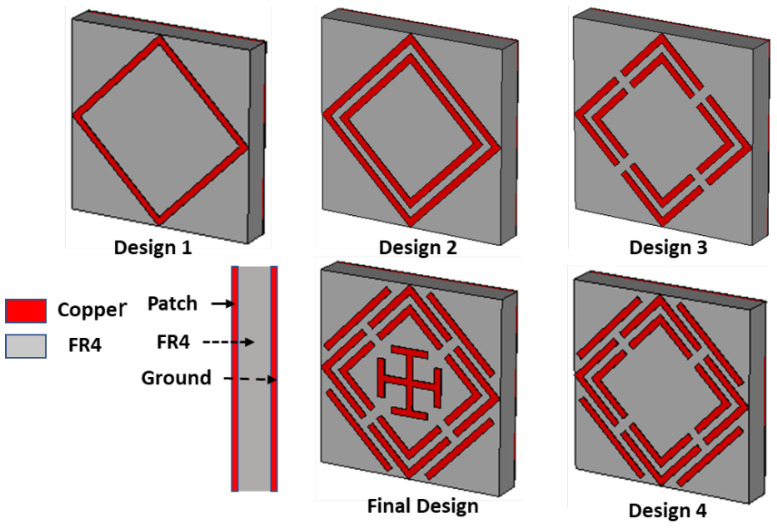
Evaluation of proposed MMA unit cell.

The evaluation of the proposed MMA towards SSRR for achieving quad-band absorption is shown in Figure 3. In order to understand the absorption mechanism of the MMA impedance, analysis is vital. The reflection coefficient (*S*_11_) depends considerably on the effective impedance $\left(Z_{e}\right)$, as shown in Equation (4).

(4) S11=Ze−Z0Ze+Z0

where $Z_{e}=\sqrt{\mu_{0}\mu_{r}(\omega )/\varepsilon_{0}\varepsilon_{r}(\omega )}$ $Z_{0}=377\Omega =\sqrt{\mu_{0}/\varepsilon_{0}}$ is the free space impedance; and $\mu_{0}$, $\varepsilon_{0}$, $\mu_{r}(\omega )$, and $\varepsilon_{r}(\omega )$ are the free space permeability, free space permittivity, frequency-dependent permeability, and permittivity, respectively. The normalized impedance can be calculated by $Z=Z_{eff}/Z_{o}=\sqrt{{\mu_{r}/\varepsilon }_{r}}$. Near-unity absorption is achieved by impedance matching with free space. The near-unity value of the real part and the near-zero value of the imaginary part represent the normalized impedance matched with free space [52,53]. The relation between the absorption and metamaterial properties can be understood by calculating Equations (3) and (4) [54].

(5) $$A(\omega )=1-S_{11}^{2}=1-{\left|\frac{Z_{e}-Z_{0}}{Z_{e}+Z_{0}}\right|}^{2}=1-\left|\frac{\sqrt{\mu_{r}}-\sqrt{\varepsilon_{r}}}{{\sqrt{\mu }}_{r}+\sqrt{\varepsilon_{r}}}\right|$$

The metamaterial attributes of the absorber are determined by the Nicolson–Ross–Weir (NRW) formula [55], where ω=2πf, and *c* is the velocity of light.

(6) εr=2ωc×d×1−(S21+S11)1+(S21+S11)

(7) μr=2ωc×d×1−(S21−S11)1+(S21−S11)

**Figure 4:**
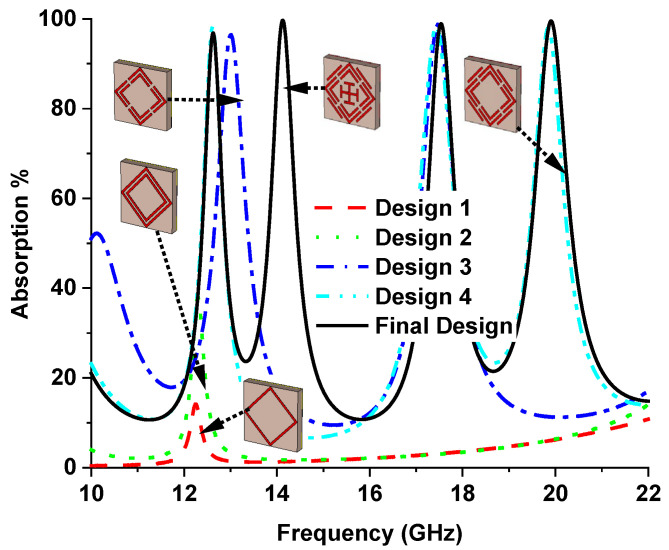
Absorption plot for different design evaluations of the proposed MMA.

**Figure 5:**
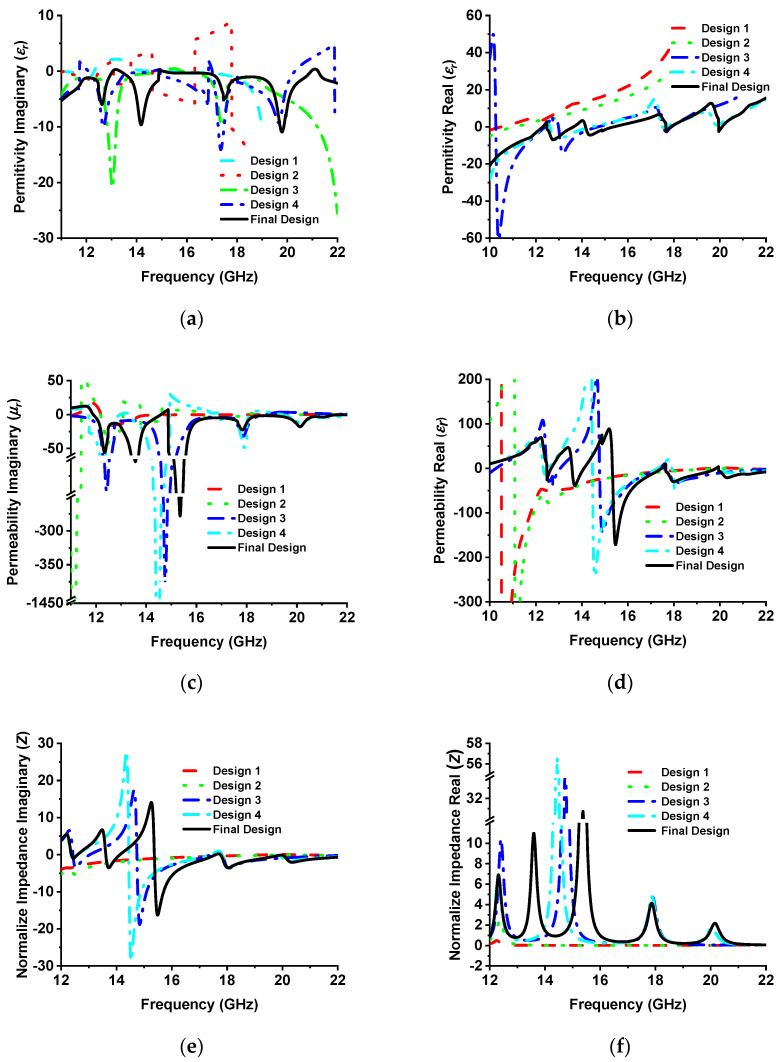
(**a**) Imaginary permittivity, (**b**) real permittivity, (**c**) imaginary permeability, (**d**) real permeability, (**e**) imaginary normalized impedance, and (**f**) real normalized impedance for different MMA designs.

Initially (design 1), a square ring resonator (SRR) is placed on top of the substrate materials, achieving only 14% absorption at a 12.25 GHz resonance frequency with (single negative) SNG metamaterial properties. The absorption percentage slightly increases to 30% at a 12.30 GHz resonance frequency using two square ring resonators because of coupling capacitance between the two SRRs, where SNG metamaterial properties are achieved. In design 3, four splits are made in the middle of each SRR, which significantly increases the capacitance in the splits, resulting in 96.37% and 98.64% absorption peaks appearing at 13.01 GHz and 17.46 GHz resonance frequency, respectively, with DNG metamaterial properties. Three absorption peaks are achieved by inserting four microstrip lines in the outer side of the SSRR. This microstrip line generates another coupling capacitance, and therefore, another absorption peak is generated. However, at 12.62 GHz, 17.42 GHz, and 19.85 GHz, SNG metamaterial properties are achieved with 97.97%, 98.23%, and 97.90% absorption peaks, respectively. Finally, a Jerusalem cross was designed at the absorber’s center to increase absorption. The absorption peaks are 97%, 99.51%, 99%, and 99.5% at 12.62 GHz, 14.12 GHz, 17.53 GHz, and 19.91 GHz, respectively. The DNG metamaterial properties appears at 12.62 GHz, 17.53 GHz, and 19.91 GHz. However, the metamaterial property is SNG at 14.12 GHz frequency. The simulated absorption plot for different designs is shown in Figure 4. Table 2 lists the metamaterial properties for various design evaluations. Metamaterial properties such as permittivity, permeability, refractive index, and normalized impedance of different designs are presented in Figure 5.

### 2.3. Equivalent Circuit Model

**Figure 6:**
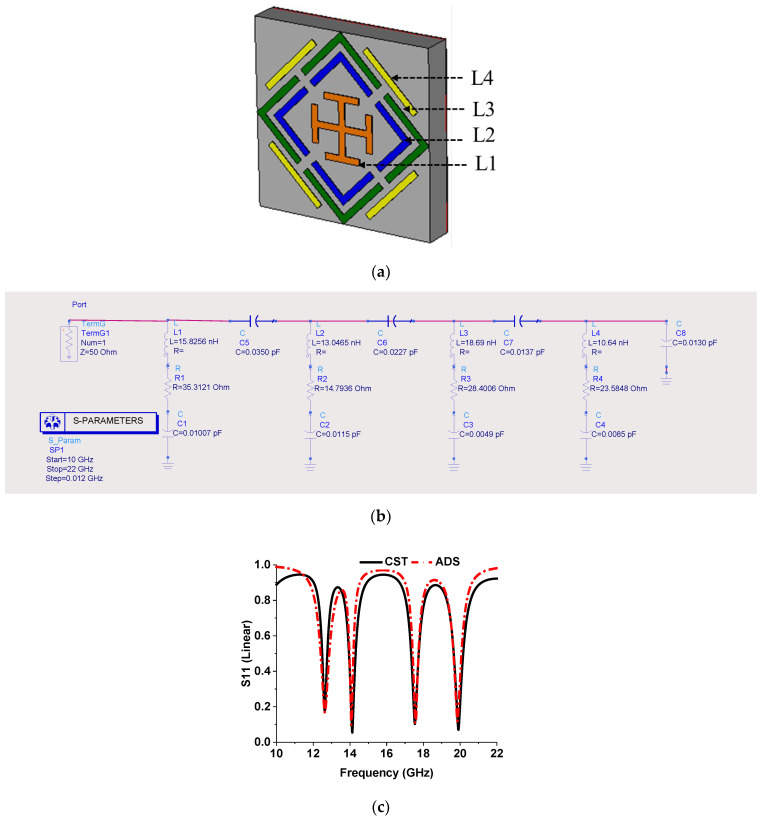
(**a**) Different resonating parts of MMA, (**b**) equivalent circuit model, and (**c**) *S*_11_ plot from ADS and CST.

The proposed MMA equivalent circuit model was designed and simulated via ADS (advanced design system) [56], as shown in Figure 6 [27,57,58,59]. Each resonant peak is constituted by the inductance and capacitance of separate elements, such as the inner Jerusalem cross, the two splits ring, and the external microstrip line, indicated different colors in Figure 6a. An RLC circuit is considered for each resonant frequency in the equivalent circuit design shown in Figure 6b. The series capacitances, C1, C2, C3, and C4, are calculated by Equation (8), where the resonant frequency is *f*, and the associated series inductance is *L* (L1, L2, L3, and L4).

(8) $$C=\frac{1}{4\pi^{2}f^{2}L}$$

The inductance (L1, L2, L3, and L4) is generated by different elements, which are calculated by Equation (9), where the substrate length is *l*; and *w* and *t* are the width of the strapline and the substrate thickness, respectively, in inches.

(9) $$L(nH)=0.00508l\left[\ln\left(\frac{2l}{w+t}\right)+0.5+0.2235\left(\frac{w+t}{l}\right)\right]$$

The coupling capacitance (C5, C6, C7, and C8) between the elements and ground is estimated by Equation (10), where *d* is the gap between the strip, and *A* represents the area of the strip. Associated series resistance is estimated by tuning for the increment and reduction in the *S*_11_ value. Figure 6c is an *S*_11_ plot of both CST and ADS simulations.

(10) $$C=\varepsilon_{0}k\frac{A}{d}$$

## 3. Results and Discussions

### 3.1. TE and TM Mode Analysis

**Figure 7:**
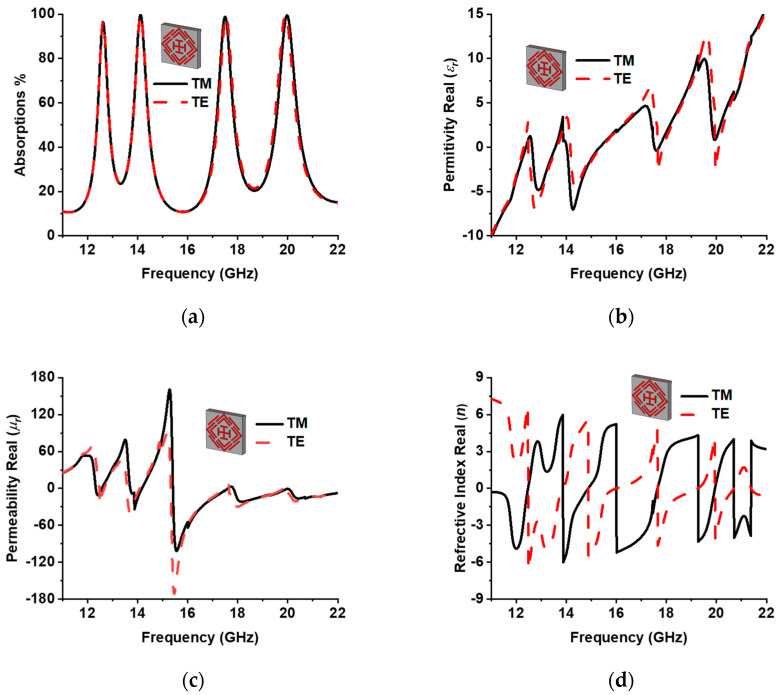
(**a**) Absorption, (**b**) permittivity (real), (**c**) permeability (real), and (**d**) refractive index (real) of the proposed MMA.

**Figure 8:**
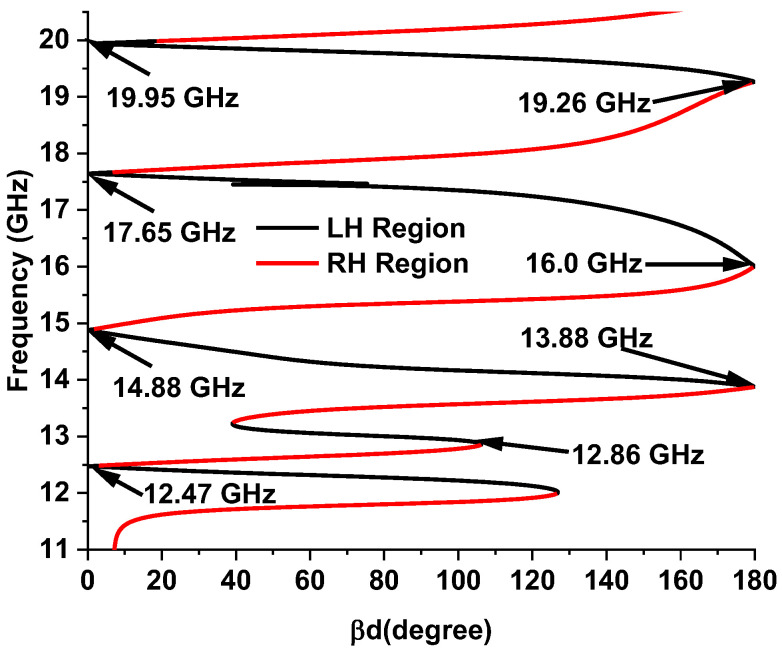
Dispersion diagram in TM mode.

The designed MMA was simulated in both TE and TM modes. Unit cell boundary conditions were applied for TM and TE simulation. Figure 7a–d presents the absorption, permittivity, permeability, and refractive index plot for all three modes, which show a feature of uniform absorption behavior. Both modes obtain the near-uniform metamaterial properties. Table 3 shows the metamaterial properties of the proposed absorber at the resonant frequency. The DNG metamaterial property appears at 14.12 GHz, 17.53 GHz, and 19.91 GHz in TM propagation mode. However, at 12.62 GHz, SNG behavior is exhibited. The dispersion diagram also validates the metamaterial properties plotted by Equation (11) [24], where d is the MMA unit cell thickness and the propagation phase constant. Figure 8 presents the dispersion curve of the designed MMA during TM mode simulation. The positive slope of the curve represents the right-hand (R) region or SNG metamaterial. The phase and group velocity are parallel in the R region. The DNG metamaterial behavior is represented by the negative slope in the left-hand (L) region, where group velocity and phase are antiparallel. In TM mode, the upper three frequencies, 14.12 GHz, 17.53 GHz, and 19.91 GHz, are located in the L region, which represents double-negative metamaterial behavior. The SNG behavior of the lower 12.62 GHz frequency is understood from the right-hand R region. The similarity of these two methods validates the metamaterial behavior of the MMA.

(11) $$\beta d=\cos^{-1}\left(\frac{1-S_{11}S_{22}+S_{21}S_{12}}{2S_{21}}\right)$$

### 3.2. Polarization Insensitivity

**Figure 9:**
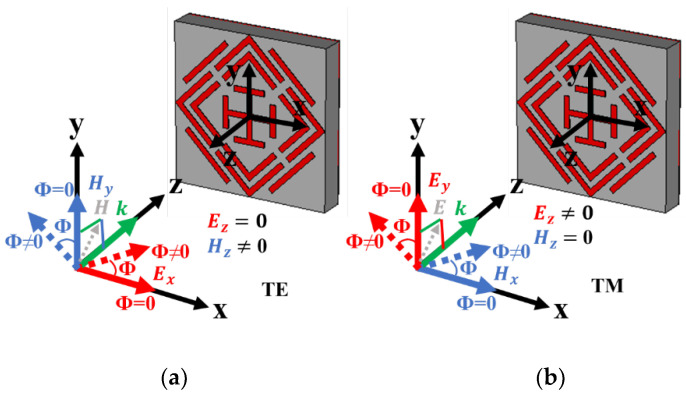
Wave vector direction in (**a**) TE and (**b**) TM mode.

**Figure 10:**
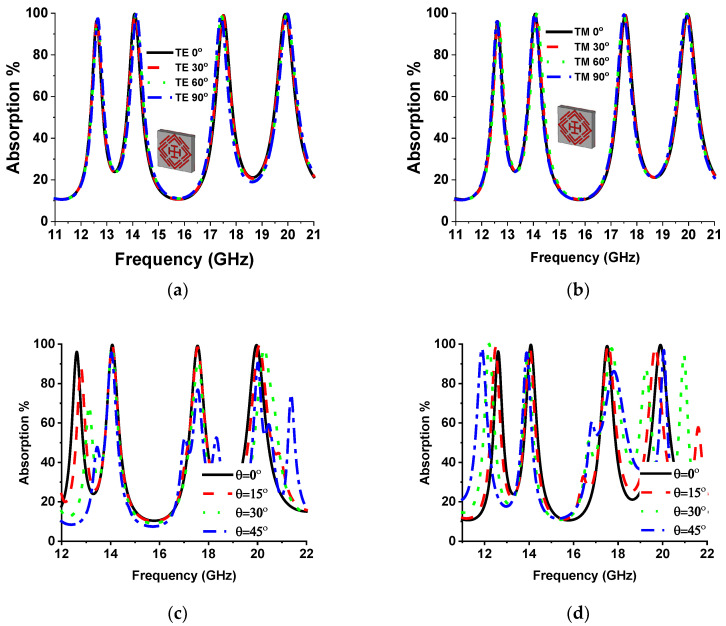
Absorption plot of different polarization angles in (**a**) TE and (**b**) TM mode and absorption plot of different oblique angles in (**c**) TE and (**d**) TM mode.

The H field ($\vec{H}$) and E field ($\vec{E}$) vector direction of the incident EM wave is presented in Figure 9a,b of the regular incident angle (θ = 0°) for TE and TM mode. The k vector towards the z-axis represents the propagation direction of the EM wave. In TE mode, there is no H vector in the z-axis, whereas no E vector exists in TM-mode propagation. Polarization-insensitive behavior of the proposed MMA for normal incident angle is plotted in Figure 10 for both TM and TE modes. The constant absorption plot for different polarization incident angles (0° to 90°) increases MMA eligibility for various applications. The reason behind the polarization-insensitive behavior is the symmetrical structural design of the proposed MMA. The designed SSRR is rotationally symmetrical, which indicates no effects on absorption at the rotation of incident EM wave vector on the XY-axis with respect to the Z-axis for circular or any other polarization of the incident wave, as shown in Figure 10a,b. Figure 10c,d shows the oblique incident angle impact TE and TM mode, respectively. In TE mode for the increment of the oblique incident angle, the absorption at 14.12 GHz shows stability up to 45°, but other resonances are either slightly shifted or reduced. On the other side, in TM mode, the absorption at both middle frequencies shows stability, whereas the upper and lower absorption peaks are shifted with the increment of oblique incident angle.

### 3.3. E-Field and H-Field Distributions

**Figure 11:**
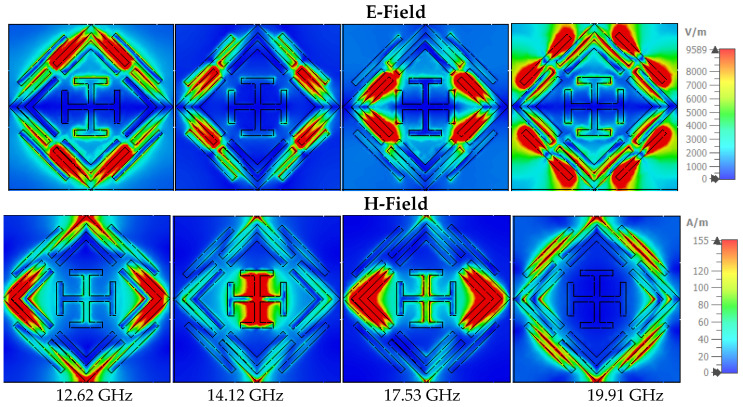
The E-field and H-field in TE mode.

**Figure 12:**
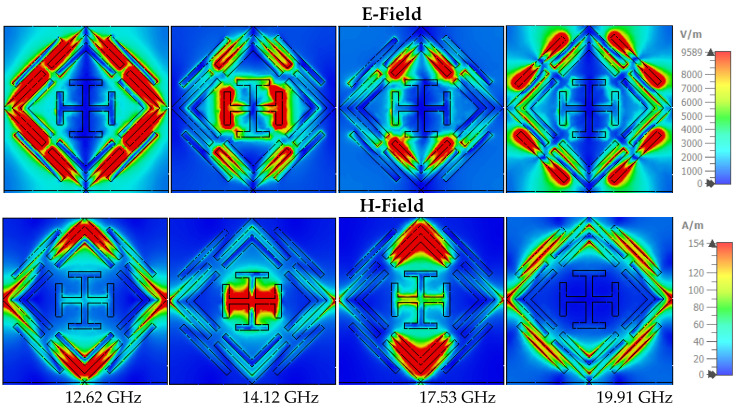
The E-field and H-field in TM mode.

The absorption mechanism can also be understood through (Magnetic field) H-field and (Electric field) E-field analysis [60]. The inter-relationship of these features can be assumed through the Maxwell equation [61,62,63]. The E-field is resonantly confined at a particular portion of the symmetrical structure. Figure 11 shows the E-field and H-field at in TE mode, where at 12.62 GHz frequency E-field is highly confined at the upper side of the external ring, and the strong H-field appears at the four corners of the outer ring. The intense magnetic field achieves absorption peaks at 14.12 GHz contributed by the vertical bar of the inner Jerusalem cross. On the other side, the E-field is strong in the left and right portions of the outer ring. The near-unity absorption at 17.53 GHz is contributed by the strong H-field of the right and left sides of the inner and outer ring, where less intensity appears in the E-field. The microstrip line on the outer ring’s external side influence the absorption peaks at 19.91 GHz. The two opposite sides of the microstrip line have an intense E-field, and the center shows high H-field intensity. Figure 12 shows the H-field and E-filed allocations in TM mode, where field intensity is similar to that in TM mode but rotated at 90 degrees.

### 3.4. Sensing Applications

**Figure 13:**
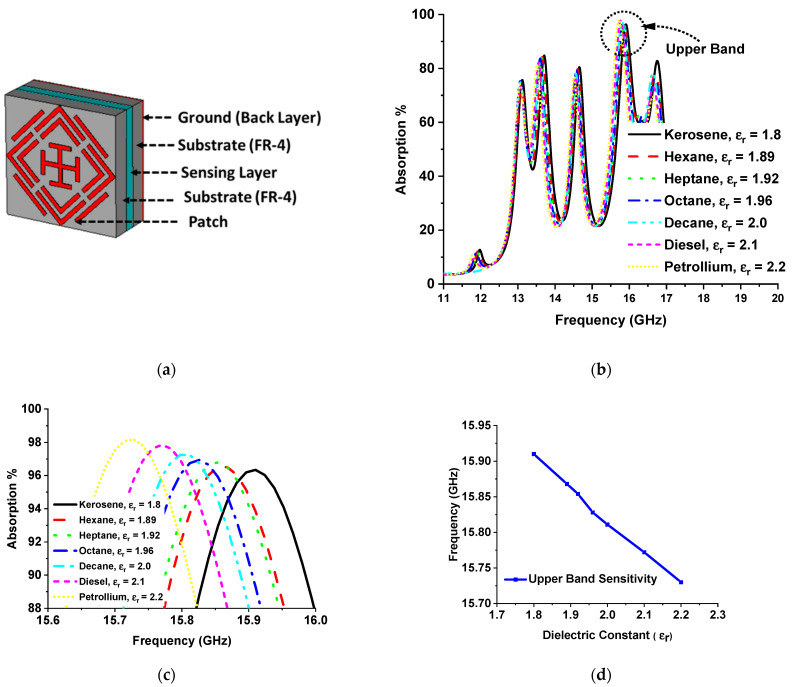
(**a**) MMA base liquid hydrocarbon sensor model; (**b**) absorption plot of different hydrocarbons; (**c**) frequency shift of different hydrocarbons; (**d**) upper-band sensitivity for liquid hydrocarbon sensor model.

**Figure 14:**
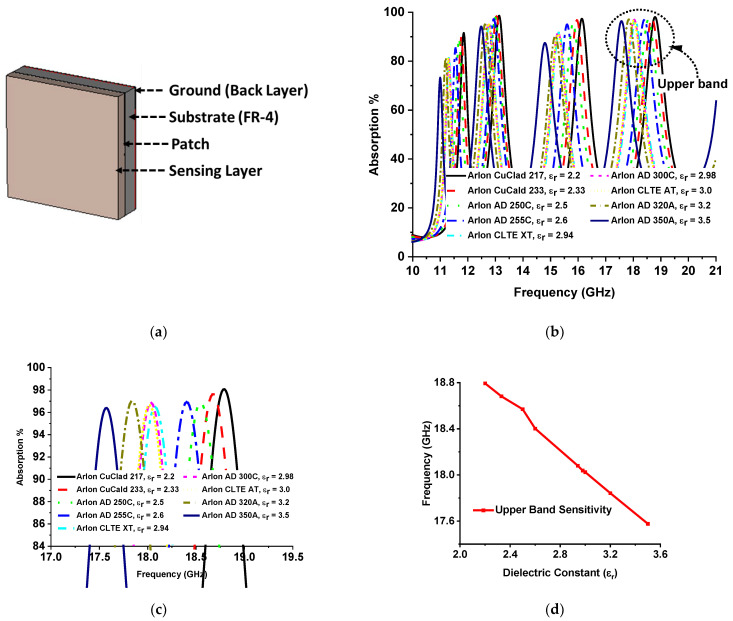
(**a**) MMA base solid material sensor model; (**b**) absorption plot of different Arlon substrates; (**c**) frequency shift of different Arlon substrates; (**d**) upper-band sensitivity for solid material sensor model.

The absorption attributes of the designed MMA depend on impedance matching, which relies on the complex value of relative permittivity and permeability. The metamaterial property can be handled by variation of the substrate thickness and dielectric property. Hence, the absorption of MMA varies with substrate thickness and dielectric constant. MMAs can be used for sensing applications in two ways: by placing a sensor layer on top of the MMA patch [34] or by placing the sensing layer between the patch substrate and substrate ground [13,30,35]. Different mechanisms of absorption-based sensor applications have been proposed from microwave to THz frequency, such as permittivity sensors [32,33], refractive index sensors [34], grin sensors [35], density sensors, temperature sensors [30], glucose sensor [64], etc. A permittivity sensing model using the proposed MMA is presented in Figure 13a. The relation between the dielectric constant and permittivity can be understood according to the equation $k=\varepsilon /\varepsilon_{0}$, where *k* is the dielectric constant, *ɛ* is permittivity, and $\varepsilon_{0}$ is the permittivity of the vacuum. The dielectric constant is the ratio of how fast an electric field travels through a material compared to a vacuum medium. For the investigation of permittivity sensing, an air gap of 1 mm is maintained between two FR-4 substrate materials as a sensing layer. The patch was designed on the upper surface of FR4 substrate 1, and no copper layer was used on the lower side. On the other hand, no copper was used on the upper side of substrate material 2, and full copper was used on the bottom side. Different hydrocarbons with individual dielectric constants were inserted in the air gap in the range of 1.8 to 2.2. The absorption curve of the MMA changes due to the overall thickness and variation of different dielectric constants of hydrocarbon that used in the sensing layer. As a result, the absorption of the lower two bands and the one upper band out of the quad band decreases. Only one absorption band shows near-unity absorption. The absorption plots for different hydrocarbon materials are shown in Figure 13b. Figure 13 shows a zoomed-in version of the absorption peaks zooming in to facilitate understanding of the resonant frequency shift with respect to the dielectric constant. The resonant frequency shifts towards a lower-frequency region with the increment of the dielectric constant by a measurable frequency interval, as shown in Figure 13d. Another permittivity sensor model for solid material sensing is shown in Figure 14a, where the sensing layer is placed on the MMA patch. Various Arlon substrate materials were chosen, with dielectric constants between 2.2 and 3.5. The integration of the sensing layer with the MMA results in a change in peak absorption due to the overall thickness and dielectric constant variation of the MMA. These changes shift the resonance frequency of the MMA, as shown in Figure 14c. The Arlon solid material sensing sensitivity is presented in Figure 14d.

### 3.5. Measurement

**Figure 15:**
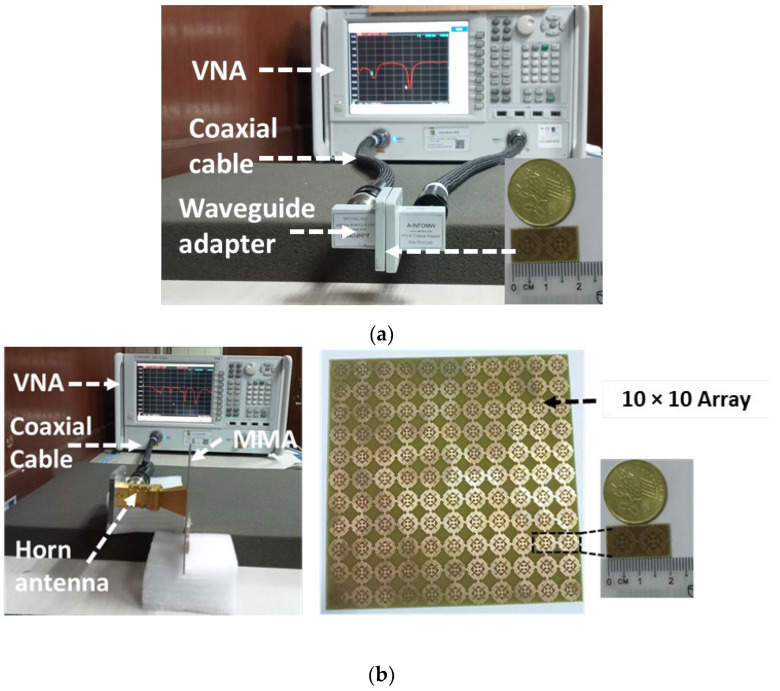
(**a**) Measurement setup with a waveguide-to-coaxial adapter; (**b**) measurement setup with a horn antenna.

**Figure 16:**
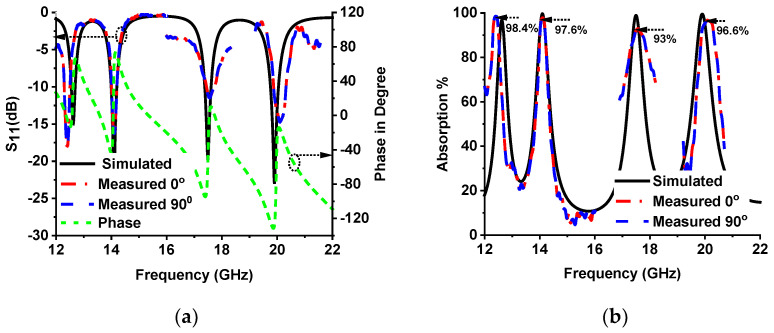
(**a**) Simulated and measured *S*_11_ (dB) with 0° and 90° polarization and simulated phase value in degree. (**b**) Simulated and measured absorption % with 0° and 90° polarization.

Figure 15 shows the measurement setup of the proposed MMA. The first three frequency bands were measured with the setup shown in Figure 15a. A vector network analyzer (VNA), coaxial cable, and waveguide to the coaxial adapter (P/N: 75WCAS, P/N: 51WCAS_Cu) and 1 × 2-unit cell prototype were used in this setup. The upper resonance frequency was measured by a horn antenna with 10 × 10-unit cells in the prototype design, as shown in Figure 15b. The agreement of the measurement and simulated values of the *S*_11_ (dB) phase in degree and absorption % are shown in Figure 16a,b, respectively. The measured absorption values are indicated in Figure 16b. The measured Q-factor is 28.83, 40.31, 13.91 and 25.76, at 12.4 GHz, 14.11 GHz, 17.56 GHz, and 20.1 GHz, respectively.

## 4. Comparison

A detailed comparative study was performed of the proposed MMA vs. existing MMAs, as shown in Table 4. Different parameters of MPA were considered, such as MPA design, size, substrate, frequency, absorption, polarization insensitivity, and applications. As discussed in previous works, an MMA that exhibits multiple absorption bands is preferable. Different MMAs were designed previously for C-, Ku-, and K-band applications. Some MMAs show absorption in other frequency spectra, such as the S and X bands. On the other side, some show polarization sensitivity, which may degrade the absorption performance at various polarization incident angles. This article represents a low-cost FR-4 substrate-based, polarization-insensitive quad-band MMA, which shows four near-unity absorption peaks in the Ku- and K-band frequencies. The proposed MMA exhibits good sensing performance for different values of permittivity.

## 5. Conclusions

In this article, we proposed a quad-band SSRR metamaterial absorber for Ku- and K-band applications. The evaluation of the MMA unit cell, impedance matching of MMA, and equivalent circuit design were discussed to understand the absorption behavior. The metamaterial property of the designed unit cell was verified by the NRW method and the dispersion calculation formula. Due to its symmetrical rotational structure, uniform absorption and polarization insensitivity has been achieved. So, the absorption performance was not verified in TE and TM simulation modes. The proposed MMA shows four absorption peaks at 12.62 GHz, 14.12 GHz, 17.53 GHz, and 19.91 GHz with absorption rates of 97 %, 99.51%, 99% and 99.5 %, respectively. The sensing performance was investigated in two modes, verifying the sensing performance of the developed MMA. Therefore, the proposed MMA is potentially appropriate for Ku- and K-band absorption and sensing applications.

## Figures and Tables

**Table 1:** Parameters list.

Parameter	Value (mm)
l	10
a1	4
a2	7.07
a3	4.95
b1	2.0
b2	1.23
g1	0.35
g2	0.35
w	0.35

**Table 2:** Metamaterial properties of different designs.

Evaluation	Peak Absorption Frequency	Permittivity (Real)	Permeability (Real)	Refrective Index	Peak Absorption
Design 1	12.25	3.62	−46.64	−0.25	14%
Design 2	12.30	2.54	−56.32	−2.57	37.84%
Design 3	13.01 17.46	−1.29 −4.44	−4.64 −2.37	−3.76 −4.71	96.37% 98.64%
Design 4	12.62 17.42 19.85	−0.39 −0.07 0.85	5.07 8.54 −2.44	1.88 3.82 −0.68	97.97% 98.23% 97.90%
Final Design	12.62 14.12 17.53 19.91	−4.51 2.88 −2.67 −2.54	−4.05 −0.93 −7.77 −6.18	−4.63 1.43 −4.63 −4.09	97% 99.51% 99% 99.5%

**Table 3:** Metamaterial properties in TM and TE mode.

Frequency (GHz)	Permittivity (Real)		Permeability (Real)		Refractive Index (Real)		Absorption %
	TM	TE	TM	TE	TM	TE	
12.62	−0.11	−4.51	5.68	−4.05	2.14	−4.63	97%
14.12	−3.81	2.88	−3.76	−0.93	−3.81	1.43	99.51%
17.53	−0.13	−2.67	−2.22	−7.77	−1.04	−4.63	99%
19.91	−0.85	−2.54	−1.59	−6.18	−0.29	−4.09	99.5%

**Table 4:** Comparison table.

Ref.	MPA	Size	Substrate	Frequency Band	Resonant Frequency		Absorption %		Polarization Insensitivity	Application
					Simulated	Measured	Simulated	Measured		
[13]	CSRR	10 × 10 × 1.6	FR-4	K	21.6 24.04	21.55 N/A	99.9% 99.9%	99.68% N/A	Yes	Absorber and sensor
[14]	Metaprogrammable analog differentiation	--	--	C	5.05-5.4	5.05-5.4	≈100%	≈100%	N/A	Absorber and analog differentiator
[24]	Wrenched square shape	10.4 × 10.4 × 1	FR-4	S, X, Ku	3.2 5.32 11.15 16.73	3.43 5.18 11.1 16.69	95.75% 95.93% 97.69% 95.64%	94.56% 96.41% 97.98% 96.67%	Yes	Absorber
[45]	SSRR	10 × 10 × 1	FR-4	S, X, Ku	3.4, 9.6, 13	≈3.3 9.6 ≈12.9	99.6, 99.1, 99.1	99.5% ≈95% 99%	Yes	Absorber
[46]	Eight identical 7-shapes	8 × 8 × 0.4	polyimide	X, Ku	8.5, 13.5, 17	8.7 14.1 17.6	99.9% 99.5% 99.9%	96% 97% 94%		Absorber
[47]	V-shaped	8 × 8 × 1.6	FR-4	Ku, K	15.52, 27.24	15.6 N/A	98.38% 90.7%	≈96% N/A	No	Absorber
[48]	T-shaped	8.5 × 8.5 × 0.2403	polyimide	Ku, K	16.77 30.92	16.85 30.79	98.7% 99.3%	98.6% 96.2%	No	Absorber
[49]	Diagonal slot patch	16 × 16 × 1.6	FR-4	Ku	12.45 14.18	12.31 13.97	99.73% 99.87%	99% 99%	No	Absorber
[27]	Fourfold resonator	9 × 9 × 1.6	FR-4	Ku	13.62 16.30	13.6 16.5	99.99% 99.99%	99.9% 99%	Yes	Absorber and sensor
Proposed	SSRR	10 × 10 × 1.6	FR-4	Ku, K	12.62 14.12, 17.53, 19.91	12.4 14.11 17.56 20.1	97% 99.51% 99% 99.5%	98.4% 97.6% 93% 96.6	Yes	Absorber and sensor

## Data Availability

The data used in this study are presented in this article.
